# What is the mechanism of tachycardia and an apparent atrioventricular nodal response during para‐Hisian pacing?

**DOI:** 10.1002/joa3.12743

**Published:** 2022-06-02

**Authors:** Takashi Kobari, Yoshiaki Kaneko, Shuntaro Tamura, Hiroshi Hasegawa, Hideki Ishii

**Affiliations:** ^1^ Department of Cardiovascular Medicine Gunma University Graduate School of Medicine Maebashi Japan

**Keywords:** accessory pathway, Coumel phenomenon, para‐Hisian pacing

A 50‐year‐old man with a history of multiple episodes of tachycardia and complete left bundle branch block (LBBB) morphology underwent electrophysiological studies and catheter ablation. The earliest retrograde activation in response to premature ventricular stimulation was observed simultaneously in the His bundle (HB) region and proximal coronary sinus (CS), without decremental conduction (Figure 1A). Retrograde conduction remained unchanged following a 20 mg bolus injection of ATP. Para‐Hisian pacing revealed an apparent atrioventricular (AV) nodal response characterized by a shorter stimulus‐atrial activation interval during the HB and ventricle capture than that during the ventricle capture alone (Figure 1B). The tachycardia observed clinically was initiated by premature atrial stimulation and immediately evolved toward a narrow QRS morphology (Figure 2A). The earliest atrial activation site during tachycardia was consistent with that of retrogradely conducted ventricular extrastimuli. However, during narrow QRS tachycardia, the His‐atrial (HA) interval was shorter, while the atrio‐His (AH) interval was longer than those measured during tachycardia with complete LBBB morphology. Moreover, premature ventricular stimuli delivered during ongoing tachycardia at the time of HB refractoriness advanced the atrial cycle and prolonged the next AH interval (Figure 2B).

**FIGURE 1:**
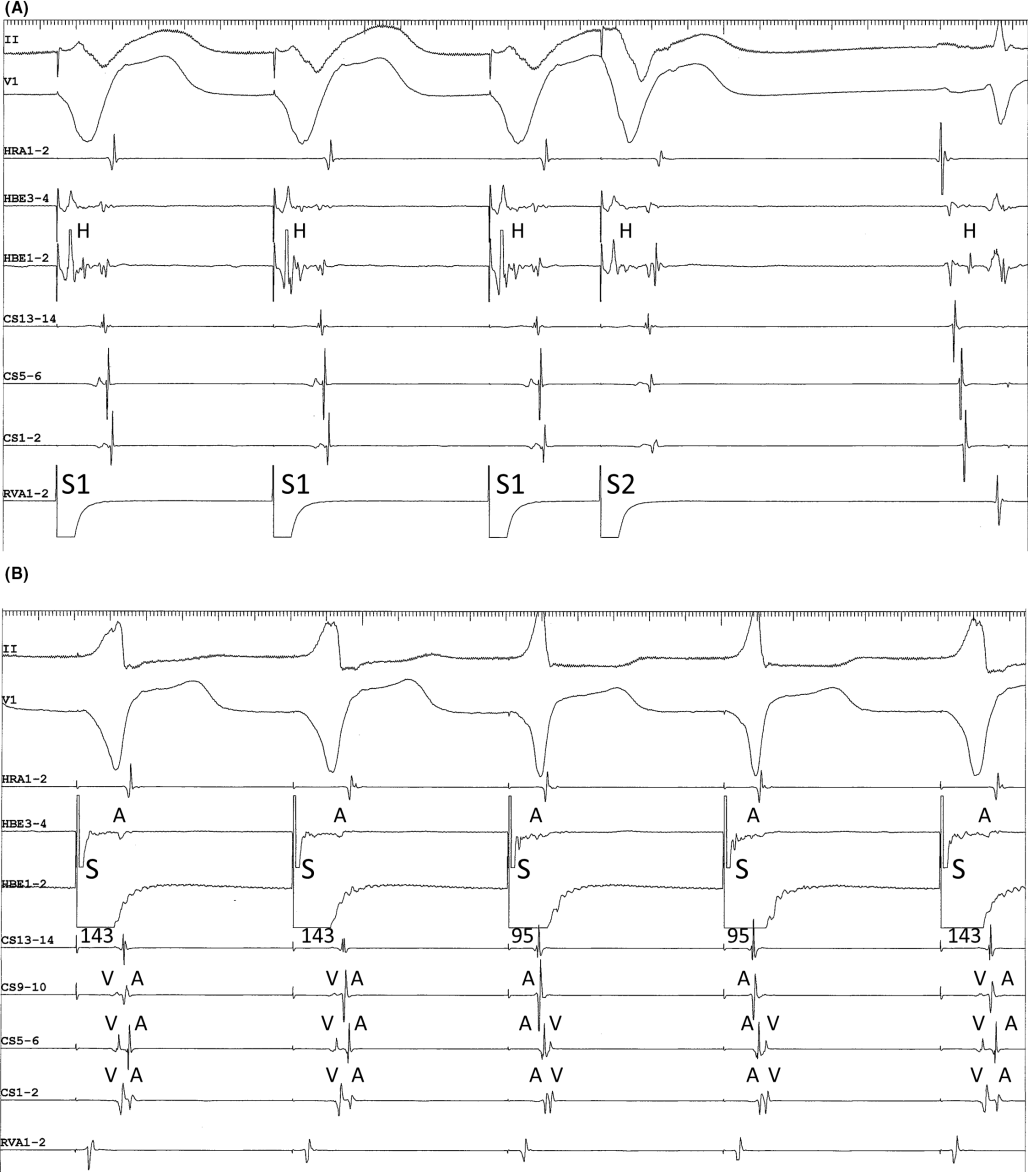
(A) Ventricular premature stimulation (S1‐S1 = 600 ms, S1‐S2 = 310 s). The His (H)‐atrial interval, including after S2, measures 47 ms. (B) Para‐Hisian pacing (S‐S = 600 ms). The QRS of the first, second, and fifth cycles are wide, consistent with direct ventricular capture, while the QRS of the third and fourth cycles are narrow, consistent with direct capture of the His bundle. The numbers correspond to the intervals between the pacing stimulus (S) and the atrial electrogram at the proximal coronary sinus (CS13‐14). A and V indicate the atrial and ventricular electrograms, respectively.

**FIGURE 2:**
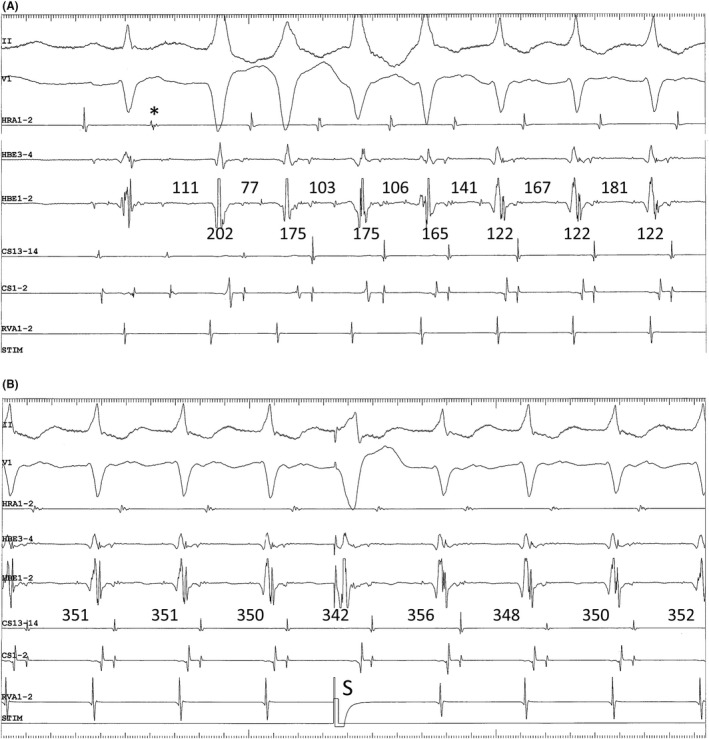
(A) Initiation of tachycardia following premature atrial contraction (*). The first four QRS have complete left bundle branch block morphologies, while the next three QRS are narrow. The numbers above and below the HBE3‐4 channel indicate the AH and HA intervals, respectively. (B) Atrial resetting of tachycardia by premature ventricular stimulus (S) delivered during HB refractoriness. The numbers above the CS13‐14 channel indicate the atrial cycle length.

Generally, shortening of the HA interval during tachycardia coinciding with the resolution of complete LBBB morphology (Coumel's law; Figure 2A), and atrial preexcitation with premature ventricular stimuli delivered at the time of HB refractoriness during ongoing tachycardia (Figure 2B), supported the diagnosis of AV reentrant tachycardia using a left‐sided AV accessory pathway as the retrograde limb of the circuit. Moreover, electrophysiological characteristics of retrograde conduction, such as concentric atrial activation and the absence of decremental properties along with sensitivity to ATP, are consistent with the presence of a left posteroseptal AV accessory pathway. A stimulus‐atrial activation interval shorter during the HB and ventricle capture than that during the ventricle capture alone may also be observed with retrograde conduction over a left posteroseptal AV accessory pathway, usually together with a shortening of the stimulus‐ventricular activation interval in the CS recordings.^1^, ^2^ However, in this case, the stimulus‐ventricular activation interval in the CS recordings remained constant, regardless of whether the HB was directly captured. Moreover, meticulous inspection revealed a transition of the earliest atrial activation site from the proximal CS during the non‐capture of the HB to the HB region during the capture of the HB (Figure 1B).

Our observations are explained as follows: during the ventricle capture alone, retrograde conduction was predominantly through the accessory pathway. In contrast, during the HB and ventricle capture, the activation wavefront propagated through the fast pathway and reached the atrium along the CS recording earlier than while propagating through the working myocardium and bundle branch and reaching the ventricular myocardium along the CS recording. Therefore, para‐Hisian pacing with direct HB capture was associated with a shorter stimulus to atrial activation interval than in the absence of direct HB capture.

Retrograde conduction over the fast pathway during para‐Hisian pacing in presence of the posteroseptal accessory pathway is often observed.^1^, ^2^ This is because the retrograde conductivity of the fast pathway may be enhanced or an unknown delay in the conduction time consumption between the pacing and accessory pathway sites may be present. Recognizing the change in the atrial activation sequence during non‐capture of HB versus capture of HB is a key clue to diagnosing retrograde conduction over the fast pathway, accompanied by retrograde conduction over the accessory pathway.^3^

The accessory pathway was successfully ablated at the left posterior mitral annulus.

## CONFLICT OF INTEREST

The authors have no conflicts of interest to disclose.

## ETHICS APPROVAL STATEMENT

Approval was obtained from the local ethics committee.

## PATIENT CONSENT STATEMENT

The patient has provided signed consent for publication.

## CLINICAL TRIAL REGISTRATION

None.
